# Role of Lipid Indices in the Assessment of Microvascular Risk in Type 2 Diabetic Retinopathy Patients

**DOI:** 10.7759/cureus.23395

**Published:** 2022-03-22

**Authors:** Devegowda Namitha, Aliya Nusrath, N. Asha Rani, Shilpashree Y Dhananjaya, Tejaswi H Lokanathan, B.N. Kruthi, A.G. Vijay Kumar

**Affiliations:** 1 Biochemistry, Adichunchanagiri Institute of Medical Sciences, Mandya, IND; 2 Anatomy, Adichunchanagiri Institute of Medical Sciences, Mandya, IND; 3 Forensic Medicine, Adichunchanagiri Institute of Medical Sciences, Mandya, IND

**Keywords:** apolipoprotein a-i & b, castelli risk index i & ii, atherogenic coefficient, atherogenic index of plasma, diabetes mellitus

## Abstract

Introduction: Diabetic retinopathy (DR) remains the leading cause of blindness and visual impairment in diabetic patients worldwide. Lipid indices (LI) such as atherogenic coefficient (AC), atherogenic index of plasma (AIP), non-high-density lipoprotein cholesterol (non-HDL-C), and Castelli risk index (CRI) I and II may be associated with bio-physiological changes of DR even when traditional lipids are within normal limit. Hence, the present study was undertaken to evaluate the LI and examine the LI predictive role in assessing the microvascular risk in diabetes patients with and without retinopathy.

Methodology: This case-control study was conducted for six months at a tertiary care hospital and included 90 subjects divided into three groups. Group I had 30 age and sex-matched healthy controls; group II and group III had 30 type 2 diabetes mellitus (T2DM) cases without DR and with DR, respectively. Plasma glucose and lipid profiles including apolipoprotein A-I (Apo A-I) and apolipoprotein B (Apo B) were measured in all subjects. LI such as AIP, AC, CRI-I, CRI-II, and non-HDL-C were calculated from the lipid profile values. ANOVA test was used to compare the means of three groups.

Results: The mean age of the study participants was 51.44 ± 11.72, 53.95 ± 12.43, and 57.16 ± 7.96 years for groups I, II, and III, respectively. Triacylglycerol (TG) showed positive correlation with all LI, AIP (r = 0.768, p < 0.00001), AC (r = 0.363, p = 0.048), non-HDL-C (r = 0.372, p = 0.042), and CRI-I (r = 0.363, p = 0.048), except for CRI-II in group III. Low-density lipoprotein cholesterol (LDL-C) showed a statistically significant positive correlation with non-HDL-C and CRI-II in diabetic subjects with and without retinopathy.

Conclusion: The study showed that LI were raised in diabetic patients with or without DR, suggesting the significant role of LI in assessing microvascular risk in T2DM, particularly when the lipid profile values seem to be normal or not disturbed markedly.

## Introduction

Diabetes mellitus (DM) is an increasing epidemic in the world today. Globally, the number of people with type 2 diabetes mellitus (T2DM) is projected to rise to 439 million by 2030, which denotes 7.7% of the world's total adult population aged 20-79 years [1]. Hyperlipidemia is the primary metabolic abnormality in T2DM that leads to complications [2]. The major complications associated with T2DM include cardiovascular diseases, neuropathy, nephropathy, and retinopathy [2].

Hyperlipidemia characterized by decreased high-density lipoprotein cholesterol (HDL-C) and increased triacylglycerol (TG), total cholesterol (TC), low-density lipoprotein cholesterol (LDL-C), and very-low-density lipoprotein cholesterol (VLDL-C) has been deliberated for modifying the risk and the severity of various metabolic disorders like hypertension, obesity, and coronary heart disease [3,4]. Based on the results of epidemiologic investigations, circulating lipoprotein species (TG, TC, LDL-C, HDL-C, VLDL-C, and apolipoproteins) are linked with the prevalence of diabetic retinopathy (DR), signifying that the pathophysiology of these lipoproteins is equivalent to that observed in cardiovascular diseases [3,4]. Apart from the traditional lipid parameters (TG, TC, LDL-C, HDL-C, and VLDL-C], increased apolipoprotein B (Apo B) and decreased apolipoprotein A-I (Apo A-I) could have greater value than the standard lipoprotein cholesterol measurement in evaluating cardiovascular risk [1].

Elevated serum lipid levels are also considered one of the strongest risk factors for retinal hard exudate pathogenic development, an early symbol of DR [5]. They are associated with an increased risk of endothelial dysfunction, which appears to play an essential role in the pathogenesis of DR, particularly concerning the dysfunctional retinal capillaries [5].

Recently, novel lipid indices (LI) such as atherogenic index of plasma (AIP), atherogenic coefficient (AC), Castelli risk index (CRI) I and II, and non-HDL-C may be associated with bio-physiological changes of DR even though the traditional lipids are within normal limit [1,2,4,6]. Therefore, LI obtained from lipid parameters reflects the proportion of atherogenic to anti-atherogenic lipid components [4]. Studies have shown dyslipidemia, including apolipoproteins, and measuring LI from traditional lipid parameters may be superior in measuring cardiovascular risk in DM patients [1,2,4,6]. Our previous study observed that lipid profiles including apolipoproteins (Apo A-I and Apo B) could be indicators of dyslipidemia in DR patients [7].

However, most of the studies have assessed the utility of LI in predicting the macrovascular complications of T2DM, and only a few studies have assessed the utility of LI in measuring microvascular complications of T2DM such as DR [2,3,6]. Against this backdrop, the present study's objective was to evaluate the LI and examine the predictive role of LI in assessing the microvascular risk in T2DM patients with or without retinopathy.

## Materials and methods

A case-control study was conducted for six months at a tertiary care hospital in south India. The study was approved by the Adichunchanagiri Institute of Medical Sciences Institutional Ethical Committee (letter number: AIMS/IEC/433/2021; dated August 14, 2021). A total of 90 study participants were selected from the ophthalmology outpatient and inpatient departments and were divided into three groups. Group I included 30 age and sex-matched healthy controls, and group II and group III included 30 cases of T2DM without retinopathy and with fundoscopic diagnosed DR, respectively. Informed consent was obtained from all the study participants, and the Institutional Ethical Committee approved the study.

Inclusion criteria

The study participants between the age group of 40-70 years were selected. Group III participants were further grouped on the basis of retinopathy grading according to the International Clinical Diabetic Retinopathy Disease Severity Scale [8]. This International Clinical Diabetic Retinopathy Disease Severity Scale describes five clinical levels of DR as mentioned in Table 1 [8].

**Table 1:** Classification of DR based on ophthalmoscopic findings. DR: diabetic retinopathy; IRMA: intraretinal microvascular abnormalities.

Stages of DR	Ophthalmoscopic findings
No apparent retinopathy	No abnormalities
Mild non-proliferative DR	Microaneurysms only
Moderate non-proliferative DR	More than just microaneurysms only but less than severe non-proliferative DR
Severe non-proliferative DR	Any of the following: (i) more than 20 intraretinal hemorrhages in each of four quadrants, (ii) definite venous beading in two or more quadrants, and (iii) prominent IRMA in one or more quadrants
Proliferative DR	One of the following: (i) retinal neovascularization and (ii) vitreous or pre-retinal hemorrhages

Exclusion criteria

T2DM patients suffering from acute and chronic inflammatory conditions (fever, bronchitis, asthma, tuberculosis, rheumatoid arthritis, psoriasis), pre-existing chronic kidney disease, history of intake of statins, history of smoking, history of chronic alcohol consumption, history of psychiatric disorders, primary hypertension, pregnant women, pre-eclamptic patients, and those with gestational DM (GDM) were excluded.

Data collection

Five milliliters of fasting and 2 ml of postprandial blood samples were drawn from study subjects under aseptic precautions. Fasting plasma glucose (FPG) and lipid profile were measured in the fasting blood sample. The postprandial blood sample was used for measuring postprandial plasma glucose (PPPG). Plasma glucose and lipid profiles in all subjects were measured using standard kits from ERBA Diagnostics (Miami, Florida) on an EM-200 auto-analyzer (ERBA Diagnostics, Miami, Florida). VLDL-C was calculated using the formula TG/5. Friedewald's formula (LDL = TC − (HDL + TG/5)) was used to calculate low-density lipoprotein (LDL). Serum apolipoproteins were measured using the turbidimetric immunoassay method using QUANTIA Apo A-I and Apo B reagents on EM-200 auto-analyzer [9].

The LI was obtained from the lipid profile values [6]. The AIP was calculated as log(TG/HDL-C), which predicts the cardiovascular risk in diabetic patients. AC was calculated as non-HDL-C/HDL-C, which measures cholesterol in LDL-C, VLDL-C, and intermediate-density lipoprotein cholesterol (IDL-C) lipoprotein fractions. CRI-I was calculated as the ratio of TC/HDL-C and CRI-II as LDL-C/HDL-C. Non-HDL-C was calculated as TC − HDL-C, indicating the lipoproteins' atherogenic component [6].

Statistical analysis

SPSS version 20 software (IBM Corp., Armonk, NY) was used for statistical analysis. Continuous variables were expressed as mean and standard deviation. The means of the three groups were compared using ANOVA and post hoc tests. Pearson's linear correlation was used to evaluate the relationship of traditional lipid parameters, and apolipoproteins with LI in T2DM patients with or without DR. Results were considered statistically significant at a p-value ≤ 0.05.

## Results

The mean age of study participants was 51.44 ± 11.72, 53.95 ± 12.43, and 57.16 ± 7.96 years for groups I, II, and III, respectively. Table 2 shows the gender distribution of subjects studied. Samples were age and gender-matched with p-values of 0.130 and 0.873, respectively.

**Table 2:** Gender distribution of subjects studied. P = 0.873 (not significant, chi-square test).

Gender	Group I		Group II		Group III	
	Number of study participants	Percentage	Number of study participants	Percentage	Number of study participants	Percentage
Female	13	43.3	12	40.0	14	46.7
Male	17	56.7	18	60.0	16	53.3
Total	30	100.0	30	100.0	30	100.0

**Figure 1:**
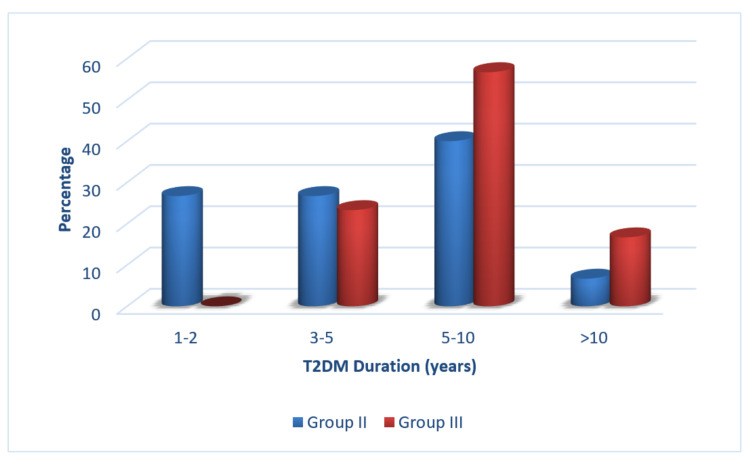
Duration of T2DM (in years) in cases (groups II and III). T2DM: type 2 diabetes mellitus.

Figure 1 shows the mean duration of diabetes in years in T2DM patients without retinopathy (group II) and DR (group III), respectively, which was significantly different (p = 0.003).

All patients with DR (group III) had non-proliferative DR (NPDR). Table 3 shows the distribution of patients with NPDR according to grade.

**Table 3:** Distribution of study participants of group III according to the grade of NPDR. NPDR: non-proliferative diabetic retinopathy.

Grade of NPDR	Number of study participants	Percentage
Mild NPDR	16	54.0
Moderate NPDR	10	33.0
Severe NPDR	4	13.0
Total	30	100.0

**Figure 2:**
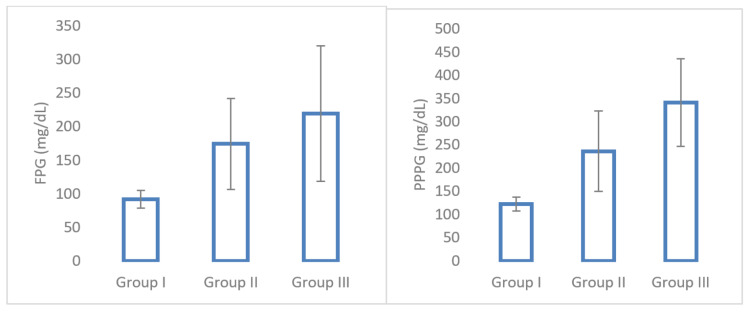
Mean values of FPG and PPPG levels among three study groups. FPG: fasting plasma glucose; PPPG: postprandial plasma glucose.

Figure 2 shows the comparison of the mean value of FPG and PPPG between the three groups, which showed a statistically significant difference (p < 0.001).

**Figure 3:**
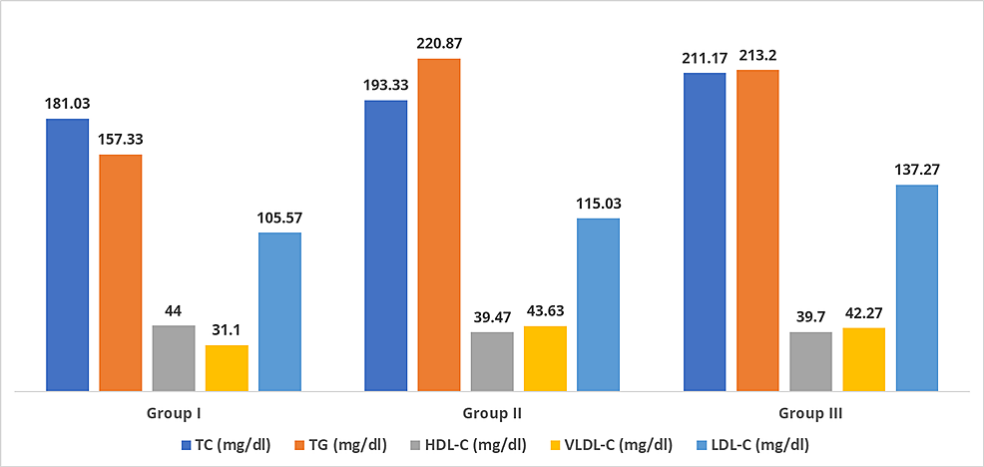
Comparison of lipid parameters among groups I, II, and III. TC: total cholesterol; TG: triacylglycerol; HDL-C: high-density lipoprotein cholesterol; VLDL-C: very-low-density lipoprotein cholesterol; LDL-C: low-density lipoprotein cholesterol.

**Figure 4:**
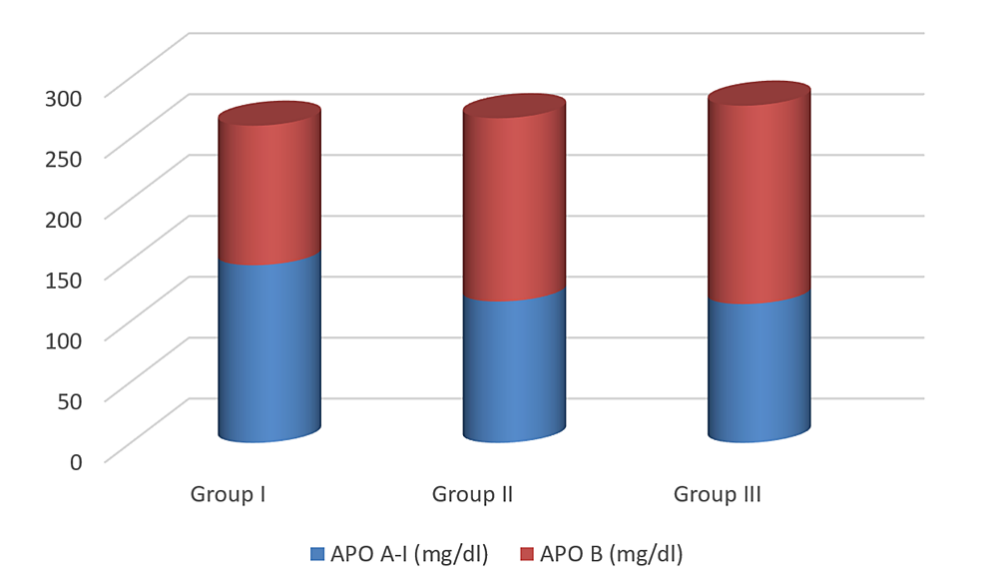
Comparison of Apo A-I and Apo B among groups I, II, and III. Apo A-I: apolipoprotein A-I; Apo B: apolipoprotein B.

Figures 3, 4 show the mean values of lipid parameters and apolipoproteins (Apo A-I and Apo B), respectively. Statistically significant alteration in the lipid profile (increased TC, TG, VLDL-C, LDL-C, and Apo B) was observed in T2DM patients with and without DR (group II and group III) compared to healthy controls (group I). The serum HDL-C and Apo A-I levels were lowered in groups II and III compared to group I subjects. The decrease in Apo A-I level was statistically significant; however, the decrease in serum HDL-C was not statistically significant in groups II and III compared to group I.

Table 4 shows the mean values of LI. LI were significantly different upon comparing these indices in both groups II and III and group I. Significant difference was seen in AIP in groups II and III compared to group I. All the other LI showed statistically significant difference only between groups I and III.

**Table 4:** Comparison of the mean value of LI in three groups studied with ANOVA and post hoc test. ** Strongly significant (p < 0.01). LI: lipid indices; AIP: atherogenic index of plasma; AC: atherogenic coefficient; CRI-I & II: Castelli risk index I & II; non-HDL-C: non-high-density lipoprotein cholesterol.

Lipid parameters	Group I	Group II	Group III	Overall p-value (ANOVA)	Significance p-value (post hoc test)		
					Group I-Group II	Group I-Group III	Group II-Group III
Atherogenic indices or LI							
AIP	0.52 ± 0.19	0.73 ± 0.18	0.72 ± 0.22	<0.001**	<0.001**	0.001**	0.965
AC	3.31 ± 1.35	4.20 ± 1.56	4.71 ± 1.89	0.004**	0.091	0.003**	0.442
Non-HDL-C	137.03 ± 35.80	153.86 ± 35.84	171.46 ± 43.84	0.003**	0.216	0.002**	0.188
CRI-I	4.31 ± 1.35	5.20 ± 1.56	5.71 ± 1.89	0.004**	0.091	0.003**	0.442
CRI-II	2.55 ± 1.12	3.13 ± 1.42	3.71 ± 1.35	0.003**	0.212	0.002**	0.195

Tables 5, 6 show the correlation between the lipid parameters and LI in T2DM patients without and with retinopathy, respectively.

**Table 5:** Pearson's correlation between lipid parameters and lipid indices in diabetic patients (group II). * Moderately significant (0.01 < p < 0.05); ** strongly significant (p < 0.01). TC: total cholesterol; TG: triacylglycerol; HDL-C: high-density lipoprotein cholesterol; VLDL-C: very-low-density lipoprotein cholesterol; LDL-C: low-density lipoprotein cholesterol; AIP: atherogenic index of plasma; AC: atherogenic coefficient; CRI-I & II: Castelli risk index I & II.

	AIP		AC		Non-HDL		CRI-I		CRI-II	
	R-value	P-value	R-value	P-value	R-value	P-value	R-value	P-value	R-value	P-value
TC (mg/dl)	0.126	0.507	0.211	0.263	0.963	<0.00001**	0.211	0.263	0.279	0.135
TG (mg/dl)	0.694	<0.00001**	0.117	0.538	0.526	0.002**	0.117	0.538	0.096	0.613
HDL-C (mg/dl)	−0.519	0.003**	−0.731	<0.00001**	0.197	0.296	−0.731	<0.00001**	−0.576	0.0008**
VLDL-C (mg/dl)	0.677	<0.0001**	0.103	0.588	0.525	<0.00001**	0.103	0.588	0.091	0.632
LDL-C (mg/dl)	0.144	0.447	0.350	0.057	0.761	0.002**	0.350	0.057	0.675	0.00004**
Apo A-I (mg/dl)	−0.433	0.016*	−0.065	0.732	−0.096	0.613	−0.065	0.732	0.120	0.527
Apo B (mg/dl)	0.0003	0.998	−0.006	0.974	0.586	0.002**	−0.006	0.974	0.086	0.651

**Table 6:** Pearson's correlation between lipid parameters and lipid indices in diabetic retinopathy patients (group III). * Moderately significant (0.01 < p < 0.05); ** strongly significant (p < 0.01). TC: total cholesterol; TG: triacylglycerol; HDL-C: high-density lipoprotein cholesterol; VLDL-C: very-low-density lipoprotein cholesterol; LDL-C: low-density lipoprotein cholesterol; AIP: atherogenic index of plasma; AC: atherogenic coefficient; CRI-I & II: Castelli risk index I & II.

	AIP		AC		Non-HDL		CRI-I		CRI-II	
	R-value	P-value	R-value	P-value	R-value	P-value	R-value	P-value	R-value	P-value
TC (mg/dl)	−0.0135	0.943	0.214	0.256	0.972	<0.00001**	0.214	0.256	0.169	0.371
TG (mg/dl)	0.768	<0.00001**	0.363	0.048*	0.372	0.042*	0.363	0.048*	0.159	0.401
HDL-C (mg/dl)	−0.790	<0.00001**	−0.780	<0.00001**	0.118	0.534	−0.780	<0.00001**	−0.718	<0.00001**
VLDL-C (mg/dl)	0.769	<0.00001**	0.367	0.046*	0.376	0.040*	0.367	0.046*	0.165	0.383
LDL-C (mg/dl)	0.144	0.447	0.155	0.413	0.733	<0.00001**	0.155	0.413	0.431	0.017*
Apo A-I (mg/dl)	−0.126	0.507	−0.035	0.854	0.148	0.435	−0.035	0.854	0.014	0.941
Apo B (mg/dl)	0.171	0.366	0.143	0.450	0.609	0.0003**	0.143	0.450	0.111	0.559

In group II subjects, TG and VLDL-C showed a positive correlation only with AIP and non-HDL-C. However, TG and VLDL-C showed a positive correlation with almost all the LI such as AIP, AC, non-HDL-C, CRI-I, and CRI-II in group III. Whereas TC, LDL-C, and Apo B showed a positive correlation with non-HDL-C in groups II and III. In addition, LDL-C showed a statistically significant positive correlation with CRI-II in diabetic subjects with retinopathy. Furthermore, HDL-C exhibited a negative correlation, which is statistically significant with all the indices in both groups II and III, except non-HDL-C.

## Discussion

The present study assessed the alterations in traditional lipid profile and atherogenic LI and their role in assessing the microvascular risk in T2DM patients.

The significant risk factors for the development of endothelial dysfunction include hyperglycemia and hypertension [2]. In this study, the mean value of FPG and PPPG was significantly elevated in groups II and III compared to group I (p < 0.001). This increase in plasma glucose levels shows that poor glycemic control is the classical risk factor for diabetic complications [10].

Lipid abnormality is the crucial cause for endothelial dysfunction and prolonged hyperglycemia and plays a prominent role in advancing vascular complications in diabetes [2,6]. The results of our study showed a significantly higher concentration of TC, LDL-C, TG, VLDL-C, and Apo B in diabetic patients with or without retinopathy than the normal healthy controls. We also found a decrease in HDL-C and Apo A-I levels, but a statistically significant reduction was present only for Apo A-I. Thus, the higher levels of TC, LDL-C, TG, Apo B, and decreased HDL and Apo A-I can lead to the formation of the hard exudate, an initial symbol of DR, attributed due to the leakage of abnormally elevated lipids from dysfunctional capillaries of the retina [7,11].

Instead of commonly measured lipid profile levels, altered LI ratios such as AIP, AC, CRI-I, CRI-II, and non-HDL-C are important in depicting better statistical association with prevalence and severity of diabetic complications [12,13]. The association between LI and diabetic microvascular complications has conflicting results. Previous studies have shown that patients with T2DM and increased lipid ratio, mainly AIP, positively correlate with microalbuminuria in T2DM and hypertension [12,13].

A study by Akdoğan et al. [14] exhibited no difference in AIP among T2DM patients with and without retinopathy. Another study by Ahmed et al. [15] studied the relationship between LI, mainly AIP, with micro and macrovascular complications in T2DM. They found that AIP levels were significantly higher in people with diabetes than controls. The present study demonstrated that LI was significantly different upon comparing these indices in both groups II and III and group I. AIP levels were statistically higher in groups II and III than healthy controls. AIP value < 0.11 is considered to be associated with a low risk of atherosclerosis [14]. AIP reveals the association between anti-atherogenic and proatherogenic lipids. AIP showed a statistically significant positive correlation with TG and VLDL-C and a negative correlation with HDL-C in groups II and III (as shown in Tables 2, 3 and Figures 2, 3). Thus, the results obtained in the present study point toward increased risk of microvascular complications in patients with elevated AIP.

AC, measured as non-HDL-C/HDL-C, accounts for cholesterol levels in LDL-C, VLDL-C, and IDL-C. It measures the overall atherogenicity among all the lipoprotein fractions [6]. In the present study, there was an increase in the levels of AC in groups II and III compared to group I. A statistically significant increase was present in group III. AC ratio positively correlated with TG and VLDL-C, particularly in group III, and negatively correlated with HDL-C. The decrease in HDL-C was statistically significant among groups II and III compared to group I. These findings indicate that the higher the LI, the greater is the microvascular risk.

CRI is calculated as a ratio of TC/HDL-C and CRI-II as LDL-C/HDL-C [6]. Similar to AC, CRI-I and CRI-II levels were increased in groups II and III compared to group I, and a statistically significant increase was present in group III. CRI-I and CRI-II showed a statistically significant positive correlation with TG, VLDL-C, and LDL, particularly in group III. Further, both CRI-I and CRI-II negatively correlated with HDL-C in groups II and III.

Non-HDL-C is the only index that measures all atherogenic Apo B containing lipoproteins and is calculated as TC − HDL-C. It is an inexpensive and potential component used as an alternate of Apo B measurement [16]. In the present study, non-HDL-C correlated positively with all the atherogenic lipoprotein components in groups II and III.

One of the strengths of the current study was that we studied the correlation between LI and microvascular complications of T2DM, unlike major studies that focused on the role of LI in macrovascular complications of T2DM. Unlike most of the studies that compared individual LI parameters with lipid profile parameters, we compared five LI (AIP, AC, non-HDL-C, CRI-I, and CRI-II) with all lipid profile parameters and found a significant positive correlation between the LI and risk of DR compared to healthy controls.

The present study had a few limitations. The first limitation was the small sample size studied. The second limitation was that we did not correlate our findings with glycated hemoglobin, a better glycemic control indicator. The third limitation was that all the DR cases were of NPDR and correlation and association were not studied between mild/moderate and severe NPDR. We wish to further our research by correlating our findings with the glycated hemoglobin in a larger sample of T2DM patients with and without DR.

## Conclusions

Our study showed a significant increase in LI in T2DM patients with DR and a highly significant positive correlation between non-HDL-C, AIP, and lipid profile parameters. Derangements in lipid profile and abnormally high LI values significantly assess microvascular risk in T2DM, especially when the absolute values of lipid profile seem to be normal or not altered distinctly.

As LI can be easily obtained from the routinely estimated lipid parameters, these indices can be used as novel markers in identifying diabetes-associated microvascular complications. Consequently, assessment of these indices can be included in addition to evaluating lipid profile alone to manage DR effectively.
